# Hirschsprung's disease - Postsurgical intestinal dysmotility

**DOI:** 10.1016/j.rppede.2016.05.001

**Published:** 2016

**Authors:** Mariana Tresoldi das Neves Romaneli, Antonio Fernando Ribeiro, Joaquim Murray Bustorff-Silva, Rita Barbosa de Carvalho, Elizete Aparecida Lomazi

**Affiliations:** a Faculdade de Ciências Médicas da Universidade Estadual de Campinas (Unicamp), Campinas, SP, Brazil

**Keywords:** Infant, Hirschsprung's disease, Gastrointestinal motility

## Abstract

**Objective:**

To describe the case of an infant with Hirschsprung's disease presenting as total colonic aganglionosis, which, after surgical resection of the aganglionic segment persisted with irreversible functional intestinal obstruction; discuss the difficulties in managing this form of congenital aganglionosis and discuss a plausible pathogenetic mechanism for this case.

**Case description:**

The diagnosis of Hirschsprung's disease presenting as total colonic aganglionosis was established in a two-month-old infant, after an episode of enterocolitis, hypovolemic shock and severe malnutrition. After colonic resection, the patient did not recover intestinal motor function that would allow enteral feeding. Postoperative examination of remnant ileum showed the presence of ganglionic plexus and a reduced number of interstitial cells of Cajal in the proximal bowel segments. At 12 months, the patient remains dependent on total parenteral nutrition.

**Comments:**

Hirschsprung's disease presenting as total colonic aganglionosis has clinical and surgical characteristics that differentiate it from the classic forms, complicating the diagnosis and the clinical and surgical management. The postoperative course may be associated with permanent morbidity due to intestinal dysmotility. The numerical reduction or alteration of neural connections in the interstitial cells of Cajal may represent a possible physiopathological basis for the condition.

## Resumo

**Objetivo:**

Descrever o caso de um lactente portador de doença de Hirschsprung na forma aganglionose colônica total que, após a ressecção cirúrgica do segmento agangliônico, manteve quadro irreversível de obstrução intestinal funcional; discutir as dificuldades no manejo dessa forma da aganglionose congênita e apontar um mecanismo patogenético plausível para o caso.

**Descrição do caso:**

O diagnóstico de doença de Hirschsprung na forma aganglionose colônica total foi definido em lactente aos dois meses de vida, após episódio de enterocolite, choque hipovolêmico e desnutrição grave. Após ressecção colônica, o paciente não recuperou a função motora intestinal que possibilitasse a alimentação via enteral. O exame do íleo remanescente pós-operatório mostrou presença de plexos ganglionares e redução numérica das células intersticiais de Cajal em segmentos proximais do intestino. Aos 12 meses de vida, o paciente mantém-se dependente de nutrição parenteral total.

**Comentários:**

A doença de Hirschsprung na forma aganglionose colônica total tem particularidades clínico-cirúrgicas que a diferenciam das formas clássicas e dificultam o diagnóstico e o manejo clínico-cirúrgico. A evolução pós-operatória pode associar-se à morbidade permanente decorrente de dismotilidade intestinal. A redução numérica ou as alterações das conexões neurais das células intersticiais de Cajal podem representar uma possível base fisiopatológica para a condição.

## Introduction

Hirschsprung's disease (HD) is the most prevalent cause of functional bowel obstruction in infants, with an incidence of 1:5000 live births.^1^ It is genetically determined and characterized by a defect in the migration of embryonic cells from the neural crest, generating an aganglionic segment at the distal end of the intestines.^2^ The gold standard diagnostic method is a rectal biopsy showing absence of ganglion cells and increased number of acetylcholinesterase-positive nerve fibers.^3^

The anatomical location of the transition between the distal aganglionic segment and the proximal ganglionic segment allows for the classification of HD as follows: classic - when the aganglionic segment extends to the proximal sigmoid; with long segment - when aganglionosis reaches the splenic flexure or the transverse colon; or total colonic aganglionosis (HDTCA) - when the aganglionic segment extends from the anus up to at most 50cm proximal to the ileocecal valve. HDTCA presents clinical, histological, and genetic differences in relation to the other types of HD, and is associated with diagnostic and management difficulties.^4^ The classic form of HD is observed in 7-88.8% of cases; the long form, in 3.9-23.7%; and HDTCA, in up to 12.6% of patients.^5^

Surgical therapy in HD minimizes the complications of intestinal obstruction when the aganglionic segment is completely resected. In some patients, postoperative intestinal dysmotility persists, most often manifested as chronic constipation and recurrent episodes of enterocolitis. Different histopathologic findings can be identified in these cases, such as incomplete resection of the aganglionic segment, hypoganglionosis, and intestinal neuronal dysplasia juxtaposed to the aganglionic zone.^4^

The present article reports a case in which difficulties of diagnosis, therapy, and prognosis were observed. The publication of this case report was approved by the Institutional Review Board (IRB) of the State University of Campinas, IRB Opinion/Article No°012/2015 from July 28, 2015.

## Case description

**Figure 1:**
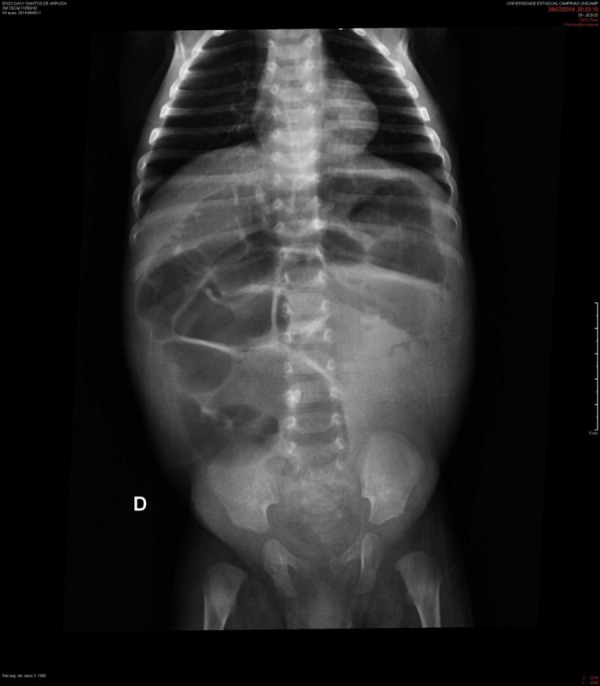
Simple abdominal radiography of a 2-month-old patient with total colon aganglionosis. Preoperative image shows dilated loops of the small bowel and colon, and low volume of air in the colon region.

A black male patient was referred to a tertiary hospital at age 2 months, with a diagnosis of intractable diarrhea and vomiting for 22 days. He evolved with hypovolemic shock and refractory metabolic acidosis. The patient had undergone a simple abdominal radiograph, which showed widespread bowel distension (Fig. 1), and a CT scan showing lack of progression of the enteral contrast to the distal colonic segments.

History of prenatal ultrasound showing distended fetal bowel loops. The mother denied delay (>24h) in the passage of meconium at birth and complaints compatible with intestinal obstruction. The child was born vaginally, at 39 weeks of gestational age, discharged from the hospital on the third day of life, with birth weight of 2950g. Neonatal screenings for phenylketonuria, hypothyroidism, and cystic fibrosis were negative. The child had been in exclusive breastfeeding since birth; the mother denied constipation, but reported poor weight gain. After 38 days of life, the child had diarrhea that required hospitalization in the city of origin. After ten days, he was transferred to a tertiary hospital for intractable diarrhea and metabolic acidosis.

Upon admission to the tertiary hospital, weighting 2860g, he presented generalized muscle atrophy, sparse subcutaneous tissue, and distended abdomen tympanic to percussion, without visceromegaly. Anus in a normal position at inspection and, at the digital rectal examination, normotonic anal sphincter and rectal ampulla of an appropriate size were observed. The patient presented explosive discharge of large amounts of liquid stool to the touch. The diagnostic hypothesis of Hirschsprung's disease was raised, and anorectal manometry was performed. At that exam, it was not possible to register rectoanal inhibitory reflex. An exploratory laparotomy was then performed; a transition zone was visualized 10cm below the ileocecal valve. A total colectomy was performed, including 2cm of ileum, followed by ileostomy. A histopathologic exam of the resected segment showed complete absence of neuronal bodies in colonic segments and scarce neuronal bodies in the myenteric plexus of the proximal ileal segment attached to the stoma.

In the post-operative period, the patient evolved with intolerance (abdominal distension and bilious vomiting) to minimum volumes of elemental formula infused via nasogastric tube. A second surgery was performed, in which the presence of bridle or kinking of the loops was discarded; 10cm of terminal ileum were resected, the fixation of the loop to the stoma was re-made, and terminal ileum biopsies were performed up to the transition with the jejunum. In these samples, ganglion cells were identified; their morphology and number were appropriate in the ganglionic plexi. In addition, samples were fixed in formalin for 48h, dehydrated in alcohol, and embedded in paraffin. Histological sections of 4µm thickness were stained with hematoxylin and eosin staining for immunohistochemical study of interstitial cells of Cajal (ICC). After heating and incubation with peroxidase, the sections were incubated with anti-CD 117 antibody, deparaffinized, placed in contact with specific antibodies, and stained. For internal control, immunohistochemical staining kits for mast cells in human neurofibroma were used. ICC were counted in ten microscopic high power fields (HPF) (400×) in the vicinity of the myenteric plexus. The mean number of ICC in surgical specimens varied widely among the observed sections, with no relation with the height in relation to the ileostomy (median: 1.2; range: 0-9.7 cells/HPF). No differences were observed in the values for each portion compared using the Mann-Whitney test (*p*=0.235), but when compared with values observed in healthy adult studies, these values were reduced.^6^

At 12 months of age, the patient persists in need of parenteral nutrition.

## Discussion

In 90% of patients, HD manifests itself in the neonatal period, characterized by delay in the first passage of meconium and progressive abdominal distension^4^ ; bilious vomiting occur in 19-37% of cases.^7^ The delay in elimination of meconium in the first 24h of life is the strongest indicator of the condition and is reported in up to 90% of patients with the disease.^7^ However, some reports have demonstrated that up to 40% of patients can eliminate meconium in the first hours of life, which indicates that this is not a required sign for diagnosis.^8^

The clinical features of HDTCA generally differ from classic forms, namely the incidence between sexes: in the classic form, it is four males to one female; in HDTCA, it is 1:1. The clinical presentation with neonatal intestinal obstruction may not be part of the clinical picture of HDACT. Late onset of symptoms (in the first weeks of life or even later, as some cases are diagnosed in adolescence) is not uncommon in this form of the disease.^9^ In HDACT, microcolon visualization on barium enema is expected; nonetheless, the colonic loops sometimes present normal size without any evidence of obstruction.^10^ Approximately 20% of patients with HDTCA undergo a second distal resection with surgical repositioning of the distal loop at the stoma, as the transition zone is irregularly arranged and difficult to be established intraoperatively.^3^ Finally, 6.5% of patients will undergo conversion into permanent ileostomy due to persistent motor dysfunction.^5^ In the case reported herein, the loop distension observed at prenatal ultrasound could have anticipated the diagnosis of intestinal obstruction, which manifested less intensely in the first days of life, perhaps due to protective effect of exclusive breastfeeding and its recognized prokinetic action and immune protection.

The occurrence of severe diarrhea in neonates is an exceptional condition in those who are exclusively breastfed and may represent evidence of serious complication from HD or another underlying disease. The enterocolitis associated with HD is observed in 50% of cases, with a higher frequency at the first trimester and first year of life, may occur prior to or after surgery.^7^ The preoperative occurrence is more common when the diagnosis is made after the first month of life.^7^ The complication manifests through abdominal distention and explosive diarrhea, which progresses to vomiting, fever, lethargy, and shock. The diarrhea is not caused by a specific intestinal infection, but by the constitutional characteristics of patients with HD. It possibly involves a relationship between the dysfunction of the enteric nervous system, abnormal production of mucin, insufficient immunoglobulin secretion, intestinal microflora unbalance, and ischemic mucosa. Therapy involves electrolytic recovery, colonic decompression, and antibiotics.^11^

The postoperative period in HDTCA is characterized by high morbidity, including food intolerance, electrolyte disturbances, and dehydration due to exacerbated intestinal secretion. Taking into account these complications prior to surgery decreases mortality, but in the long run, there is greater risk of stoma permanence for long periods in these patients.^12^ In the present case, the analysis of the proximal segment indicated hypoganglionosis, characteristic of the transition zone, which may explain the need for a second intervention.

Molecular biology and pathology anatomy techniques in samples from patients with intestinal neuropathies indicate that inflammatory and degenerative aggressions resulting from ischemia, luminal stasis, and changes in the microbiota may compromise the morphofunctional integrity of the enteric nervous system and of other elements responsible for the intestinal motility, and may result in postoperative morbimortality.^4^

Quantitative changes or the network connections of the ICC have been described in various conditions that evolve with gastrointestinal dysmotility, such as esophageal achalasia, gastroschisis, intestinal pseudo-obstruction, necrotizing enteritis, inflammatory bowel disease, and anorectal malformation.^13^

The ICC are developed from embryonic mesoderm and are located between the nerve fibers and smooth muscle cells of the intestinal wall. These cells are capable of transmitting impulses from the enteric motor neurons and of generating rhythmic electrical slow wave activity. They coordinate the pacemaker activity and propagation of slow waves, and act in the sensitive identification of muscle stretch phenomena. In the gastrointestinal tract, they are distributed from the upper esophageal sphincter to the internal anal sphincter,^13^ juxtaposed to the nerve endings of myenteric motor neurons. The presence of the tyrosine kinase receptor on the cell membrane allowed the development of anti-c-kit antibodies for the histological identification of these cells.^14^ The absence or reduction in the number of ICC determines slow-wave abnormalities, causes decreased contractility of the smooth muscle cells, and decreases the transit rate of the intestinal content.^15^

In the aganglionic intestine and in the HD transition zone, there may be a reduced number and/or disturbances in the communication network of c-kit positive ICC.^16^ Few studies have assessed the relationship between HD and the distribution of ICC in the remaining ganglionic segment. One study of these cells in the remaining intestinal ganglionic segment of 15 children identified normal count in 13 cases, which have evolved without postoperative dysmotility. In two cases, however, the histopathologic study indicated a significant reduction in the ICC count, and the patients developed severe and persistent constipation, necessitating further resection of the remaining dilated segment.^17^ In another series of cases, the ICC count in the colon ganglionic 11 children with HD was compared with the count in the control group. One patient had a reduced count and developed severe constipation.^18^ Gfroerer and Rolle reviewed the case reports available and concluded that there is considerable heterogeneity in the ICC expression in the gut-associated lymphoid tissue of patients with HD, and that the different patterns of expression could result from differences in the size of aganglionic segments to aganglionic segment resection, as well as from differences in cell-counting methods.^19^

These findings require careful interpretation, given the scarcity of studies that associate ICC count and motor function in humans. Furthermore, the examined tissue samples were from patients with obstructive disease during different periods, which makes it difficult to determine whether the reduction is a consequence of the obstruction process or a pathogenetic association with HD. The diversity of the ICC defects may indicate that this phenomenon is secondary to changes resulting from intestinal obstruction, as the ICC are particularly sensitive to ischemia.^14^^,^ 15 The inability to visualize all cells from formalin-fixed paraffin samples is an additional factor that hinders the definition of a histopathological diagnosis.^19^

In the present patient, it was not possible to compare the histological sample with those of healthy children, a scarcely available material.^20^ Appropriate tissue samples, correct handling, and expertise in anatomopathological interpretation are crucial to increase the accuracy of diagnosis of low ICC count. It would be worthwhile to assess a larger number of patients with unfavorable outcomes to determine the significance of the histopathological finding of decreased ICC and its correlation with the clinic presentation.
